# Frequency of surgical bone augmentation methods complementary to dental implant placement: A study evaluated with cone beam computed tomography

**DOI:** 10.4317/jced.61148

**Published:** 2023-12-01

**Authors:** Gutierrez-Draxl Mateo, Juan-Pedro Mazón-Esteve, Carlos-Rafael Pineda-Villacorta, Juan-Antonio Blaya-Tarraga, José-María Diaz-Fernandez

**Affiliations:** 1 Universidad Europea de Valencia. Faculty of Health Sciences. Department of Dentistry

## Abstract

**Background:**

The success of dental implants largely depends on the quantity and quality of available bone. Occasionally, it is necessary to perform additional surgical techniques alongside implant placement to increase the available bone volume and ensure the success and survival of treatments. The objective of this study was to evaluate, through cone beam computed tomography, the need for supplementary bone augmentation methods in implant placement. Additionally, the study aimed to assess the frequency of such techniques based on gender, anatomical sectors, and types of bone augmentation procedures.

**Material and Methods:**

The analysis included 106 cone beam computed tomography images obtained from 77 patients over the age of 18 who sought oral rehabilitation with implants at the University Clinic of the Master’s Program in Oral Implantology at the European University of Valencia.

**Results:**

A total of 201 edentulous sextants were analyzed. It was observed that 63.68% of the sextants required a bone augmentation technique, and there was a statistically significant difference (*p*=0.039) regarding the need for supplementary techniques in women. The need for bone augmentation by sectors was most prevalent the horizontal type (48.11%) and in the mandible (29.41%). About crestal and lateral approaches for sinus elevation, there was a higher need for the lateral technique (49.38%), and a statistically significant difference was evident (*p*=0.015).

**Conclusions:**

A high frequency of bone augmentation need for implant placement was demonstrated. It was shown that some form of supplementary surgical method was required in implant placement (63.68%). The highest need for bone augmentation was observed in the posterior maxillary sector, primarily in the vertical type (29.27%), accompanied by lateral window sinus elevation technique (49.38%).

** Key words:**Bone graft, Dental implant, Guided bone regeneration, Sinus floor augmentation, Cone beam computed tomography.

## Introduction

The success of dental implants largely depends on the quantity and quality of available bone in the jaw or maxilla. Ideally, the implant should be surrounded by bone, and the distance between the vestibular wall and the implant (PV-I) should measure between 1 and 2 mm to ensure adequate support (1,2). It is also advisable to consider the bone resorption phase, so a PV-I distance of at least 4 mm is recommended for immediate implants to ensure the maintenance of a 2 mm thickness of the vestibular plate after the osseointegration phase (3).

To achieve these parameters and simultaneously get an ideal surgical and prosthetic positioning of the implant, it is occasionally necessary to perform supplementary procedures involving some form of graft or additional technique to enhance the size and/or density of the area to be rehabilitated. Among the most common surgical methods are guided bone regeneration (GBR), block bone grafting (BBG), maxillary sinus lift, and alveolar preservation (4). Additionally, techniques like the Split Crest or osteogenic distraction may be employed.

Currently, the use of cone-beam computed tomography (CBCT) has become a diagnostic and planning tool in various branches of dentistry (5). CBCT allows us to reduce complications and enhance surgical safety as it enables us to pre-evaluate available bone volume and select the appropriate implant dimensions (6). According to Carter *et al*., more than 59% of private practices utilize CBCT in routine preoperative planning for dental implants as a pre-surgical assessment of the need for complementary surgical techniques (7).

Assessing the frequency of the need for supplementary surgical methods alongside dental implant placement is a crucial aspect of clinical practice, as well as in the research and development within the field of dentistry at the university level. Therefore, this study aims to quantify the need for additional bone augmentation (BA) techniques during the placement of implants in edentulous sextants. Likewise, it seeks to evaluate whether there is a gender-based difference in the need. Additionally, it will explore whether there is a greater need in the maxillary or mandibular sectors. Similarly, it will assess which areas require horizontal, vertical, or combined bone augmentations the most, and determine how many sextants require sinus lift with crestal or lateral techniques in patients seeking implant-prosthetic rehabilitation in the Master’s program in Implants at the European University of Valencia.

## Material and Methods

- Study Design and Sample

This study followed a retrospective cross-sectional design. The analysis of cone-beam computed tomography (CBCT) images was conducted on patients aged 18 and older who sought oral rehabilitation with dental implants at the European University in Valencia from September 2022 to March 2023. The collected sample size comprised 77 patients from whom 106 CBCT scans were obtained, encompassing 201 edentulous sextants.

- Ethical Considerations

The study received approval from the European University`s Research Committee (Ref. CIPI/23.163) (Annex 1). Additionally, the collection of tomographs was authorized by the Director of the Clinical Department of Dentistry (Annex 2).

- Collection of Cone-Beam Computed Tomography Scans

The entire sample was collected at the University Dental Clinic of the European University in Valencia. CBCT scans were taken from patients aged 18 and older who came in for implant evaluations and had at least one sextant with a missing tooth. Edentulous patients were excluded from the sample.

- Implant Planning and Data Recording

Data collection commenced by exporting all CBCT scans that met the selection criteria of the University Clinical Center. Once the sample was gathered, analysis was conducted by the same single individual in an environment with minimal noise and external light, utilizing a laptop with a screen resolution of 1920 x 1080 to optimize image quality.

Implant planning was carried out using “BlueSkyPlan®” software from Blue Sky Bio, which allowed for subsequent determination of which implants would require bone augmentation techniques through direct measurements. Information was recorded using a data collection form, assigning a unique number to each form and entering patient data.

Once the image was loaded into the program, a panoramic curve was traced from the axial plane, edentulous areas were identified, and prosthetic planning was performed. Implant positions were determined accordingly. Following the schema proposed by Al-Johany *et al*. (8), a standard diameter ranged from 3.75 mm to less than 5 mm, and a standard length ranged from 10 mm to less than 13 mm. Consequently, implants of 4 mm x 10 mm were planned, ensuring a minimum distance of 2 mm between the implant and adjacent teeth, and 3 mm in the case of two adjacent implants (9). From a sagittal view, subosseous implants were positioned, and if possible, a minimum of 2 mm distance between the implant and the vestibular bony wall was maintained (2,3). Additionally, in mandibular cases, the inferior alveolar nerve pathway was traced, and at least 1 mm distance between the nerve and the implant was observed (10). Taking this into account, horizontal bone regeneration was indicated if the vestibulo-palatal/lingual distance was less than 7 mm or, in cases with sufficient width but necessitated due to prosthetic positioning of the future crown and bone defect, bone augmentation was deemed necessary. The ABC classification of Wang *et al*. (11) was employed to determine the need for vertical bone regeneration when the bone-to-amelocemental line distance exceeded 3 mm. In cases where both horizontal and vertical defects were observed, combined bone regeneration was indicated. Likewise, the ABC classification was used to determine the need for sinus lift and whether it should be performed using a crestal or lateral approach, based on residual bone height of at least 6 mm, or a lateral window approach if otherwise.

- Data Analysis

Data analysis was conducted using RStudio 4.3.0® statistical software. Firstly, proportions of categorical variables were calculated. Subsequently, the association between the need for additional techniques by sextants and the comparison of sinus lift necessity were determined using the Chi-square test. Lastly, Fisher’s Exact Test was used to associate different types of bone augmentations with posterior and anterior sectors.

## Results

A total of 106 cone-beam computed tomography scans from 77 patients were selected and analyzed, encompassing 201 edentulous sextants.

Table 1 presents the sociodemographic characteristics of the study population. 51.95% of the scans were from male patients, and the overall median age was 59 years. Regarding tomographic characteristics, it was observed that 128 (63.68%) of the sextants required a bone augmentation technique for implant placement.

**Table 1:**
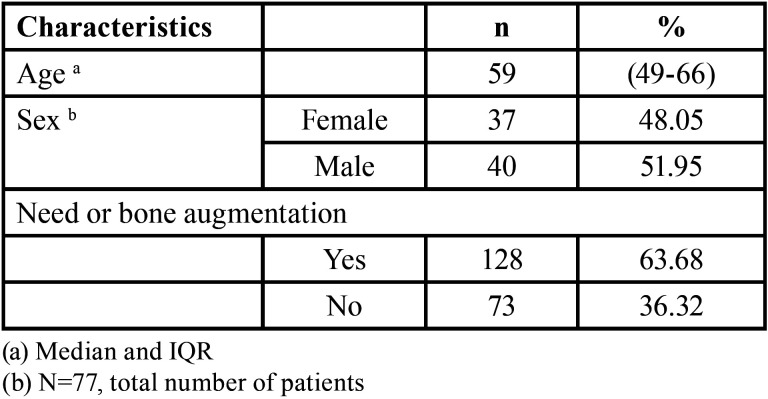
Tomographic and Sociodemographic Characteristics of the Study Population. (N=201 edentulous sextants).

In Table 2, sextants 1-3 and 4-6 are grouped, resulting in 4 subgroups: posterior maxilla, anterior maxilla, posterior mandible, and anterior mandible. The aim was to establish the relationship between tomographic and sociodemographic characteristics and the need for bone augmentation techniques. When comparing by gender, it was found that out of 95 sextants from female patients, 68 (71.58%) required some form of bone augmentation, while 27 sextants (28.42%) did not. The associated *p-value* for this relationship is 0.039, indicating a statistically significant difference between genders in terms of the need for bone augmentation. When comparing sectors, it is observed that the area with the highest need for bone augmentation techniques is the posterior maxilla, with a total of 69 sextants. In contrast, the anterior mandible has the lowest need, with only 4 edentulous areas recorded. Finally, the need for bone grafts was compared between the anterior and posterior sectors, both upper and lower, obtaining *p-value*s of 0.566 and <0.001, respectively, with the latter being statistically significant.

**Table 2:**
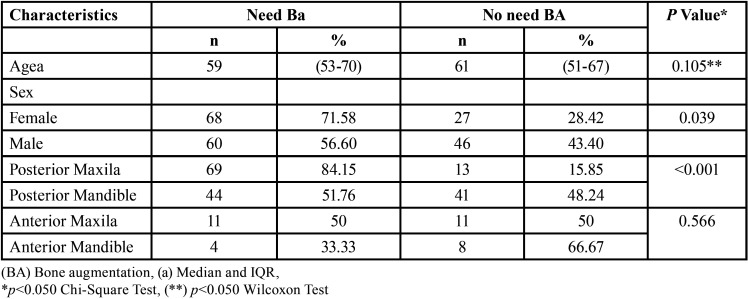
Relationship between participant characteristics and edentulous sextants with the need for any bone augmentation technique (N=201).

In Table 3, a detailed analysis of the frequency of each type of bone augmentation in both the posterior and anterior sectors was performed. It was found that horizontal bone augmentation was the most prevalent type, accounting for 48.11% of the total cases. Second in prevalence was vertical augmentation at 26.42%, followed by combined augmentation at 25.47%.

**Table 3:**
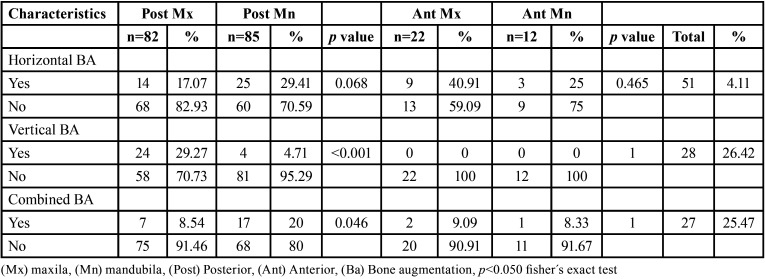
Comparison of the need for different types of bone augmentations between posterior and anterior sextants.

When comparing the need for horizontal bone augmentation in the posterior sectors, it was observed that the mandible required a higher percentage of bone augmentations, at 29.41%, while in the maxilla, it was 17.07%. Similarly, combined bone augmentation was found to be necessary in 20% of the lower posterior edentulous areas, compared to 8.54% in the upper areas. This difference proved to be statistically significant (*p*=0.046). In contrast, vertical bone augmentations were more prevalent in the posterior sectors of the maxilla, representing 29.27%. Furthermore, this difference was statistically significant, with a *p-value* of 1.86e-05.

On the other hand, in the anterior sectors, bone augmentation, besides having a small sample size, showed no statistically significant differences in any of the comparisons between sextant 2 and sextant 5.

In Table 4, a comparison between crestal and lateral sinus lift procedures in the posterior sector was conducted. It was observed that crestal sinus lifts would be necessary in 24 (29.63%) edentulous sextants, while lateral window lifts in 40 (49.38%). This difference yielded a *p-value* of 0.015, indicating statistical significance.

**Table 4:**
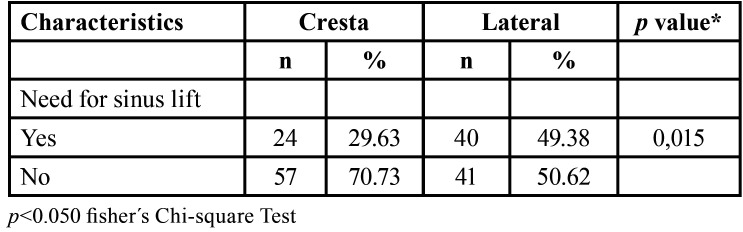
Comparison of the need for crestal and lateral sinus lift in the posterior maxilla (N=81).

## Discussion

The purpose of this study was to evaluate the frequency of the need for surgical bone augmentation methods in addition to implant placement. Currently, implant placement is generally determined based on restorative needs, which implies that multiple methods are performed to increase deficient alveolar bone and allow implant placement to accommodate the desired prosthesis (1).

For the analysis of variables, cone-beam computed tomography scans were used in patients who came for dental implant placement studies. Although implants have been used predictably and with high success rates in clinical practice for many years using different imaging techniques, three-dimensional (3D) radiography using CBCT has become an established diagnostic technique in various dental applications, including dental implant surgery (12). This is because, in addition to bone dimensions and volumes, it provides precise data about anatomical structures, bone defects, anatomical variations, pathologies, etc (13).

When evaluating the frequency of the need for complementary surgical methods in the study population, it was observed that at least one technique was necessary in 63.68% of the total edentulous sextants. This result is similar to the one found in the study by Bornstein *et al*., (12) where out of a total of 1368, 60% required some type of bone augmentation procedure. However, other studies like those by Cha *et al*. and Caracaș *et al*. (14,15) reported lower percentages of 50.3% and 43.33%, respectively. The difference in results may be due, on the one hand, to the fact that the results of this study were based on planning with only 4x10 mm implants, while Cha *et al*. and Caracaș *et al*. do not specify the dimensions of the implants, which may mean that they used some shorter and narrower ones to avoid additional treatment. On the other hand, Ratnayake *et al*. (16) point out that there is an annual increase of 13% in bone graft procedures, including those in dentistry.

In this study, it was evident that the need for complementary surgical techniques in women (71.58%) was higher compared to men (56.60%), with a statistically significant difference between genders (*p*=0.039). This aligns with studies such as Caracaș *et al*., (14) which reported a higher frequency (61.5%) of biomaterial use in female cases. This could be attributed to the difference in bone development between women and men. Men reach their peak bone mass growth after women, which allows them to have higher bone density. Once this stage is reached, cortical thickness in women is similar or slightly greater. However, periosteal apposition continues in both sexes, being more noticeable in men, while endosteal resorption is more predominant in women. As a result, over time, women tend to experience bone loss at an earlier age and at a faster rate (17-19).

The total number of cases, both maxillary and mandibular, requiring vertical bone augmentation was 28, of which 85.71% were required in the maxilla. This difference was statistically significant. Similar results were found in the studies by Urban *et al*. and Funato *et al*. (20,21), with a prevalence of vertical GBR in the maxilla at 85% and 73.68%, respectively.

When comparing the need between the two types of sinus lift approaches, a statistically significant difference was evident (*p*=0.015). Lateral sinus lift was necessary in 49.38%, while the indirect approach was only required in 29.63%. In a systematic review of the frequency of sinus anatomical variations, Ata-Ali *et al*. (22) mentioned that before the presence of septa, sinus pneumatization is the most prevalent anatomical variation at 33.2-58%, with increased osteoclastic activity in the periosteum of the Schneider membrane causing sinus expansion. Additionally, it is believed that additional positive pressure contributes to alveolar bone atrophy. In the posterior maxilla, soft type IV bone has little resistance to these processes. As a result, vertical alveolar bone height decreases in edentulous areas (23). This could be the reason why there is less residual bone in the posterior edentulous sectors and fewer crestal lifts are required compared to lateral lifts.

## Conclusions

In this study, it was determined that at least one complementary technique to implant placement was required in 63.68% of the evaluated edentulous sextants. Likewise, the gender distribution was not balanced, as women (71.58%) have a predisposition over males (56.60%) for the need for bone augmentation.

The area that most required any of the complementary techniques was the posterior maxilla in 84.15% of the evaluated edentulous zones. Furthermore, these were more frequent in the maxilla than in the mandible.

The need for horizontal bone augmentation was the most prevalent at 48.11% compared to other forms of bone regeneration. Vertical AOs were more often registered as necessary in the maxilla. On the contrary, combined bone augmentation was more prevalent in the mandible.

In the evaluation of the posterior sectors of the maxilla, a greater need for lateral sinus lift (49.38%) was evident compared to the crestal approach (29.63%).

## References

[1] Doonquah L, Holmes PJ, Ranganathan LK, Robertson H (2021). Bone Grafting for Implant Surgery. Oral Maxillofac Surg Clin North Am.

[2] Nunes LS de S, Bornstein MM, Sendi P, Buser D (2013). Anatomical characteristics and dimensions of edentulous sites in the posterior maxillae of patients referred for implant therapy. Int J Periodontics Restorative Dent.

[3] Capelli M, Testori T, Galli F, Zuffetti F, Motroni A, Weinstein R (2013). Implant-Buccal Plate Distance as Diagnostic Parameter: A Prospective Cohort Study on Implant Placement in Fresh Extraction Sockets. J Periodontol.

[4] Cha HS, Kim JW, Hwang JH, Ahn KM (2016). Frequency of bone graft in implant surgery. Maxillofac Plast Reconstr Surg.

[5] Wu X, Cai Q, Huang D, Xiong P, Shi L (2022). Cone-beam computed tomography-based analysis of maxillary sinus pneumatization extended into the alveolar process in different age groups. BMC Oral Health.

[6] Deeb G, Antonos L, Tack S, Carrico C, Laskin D, Deeb JG (2017). Is Cone-Beam Computed Tomography Always Necessary for Dental Implant Placement?. J Oral Maxillofac Surg.

[7] Carter JB, Stone JD, Clark RS, Mercer JE (2016). Applications of Cone-Beam Computed Tomography in Oral and Maxillofacial Surgery: An Overview of Published Indications and Clinical Usage in United States Academic Centers and Oral and Maxillofacial Surgery Practices. J Oral Maxillofac Surg.

[8] Al Johany SS, Al Amri MD (2017). Alsaeed S, Alalola B. Dental Implant Length Diameter: A Proposed Classification Scheme: Implants Classification by Length and Diameter. J Prosthodont.

[9] Phillips DJ, Swenson DT, Johnson TM (2019). Buccal bone thickness adjacent to virtual dental implants following guided bone regeneration. J Periodontol.

[10] Amaral CA, Freitas M, Joly JC (2020). Guided bone regeneration in staged vertical and horizontal bone augmentation using platelet-rich fibrin associated with bone grafts: A retrospective clinical study. Int J Implant Dent.

[11] Wang HL, Katranji A (2006). ABC sinus augmentation classification. Int J Periodontics Restorative Dent.

[12] Bornstein MM, Brügger OE, Janner SF, Kuchler U, Chappuis V, Jacobs R (2015). Indications and Frequency for the Use of Cone Beam Computed Tomography for Implant Treatment Planning in a Specialty Clinic. Int J Oral Maxillofac Implants.

[13] Ataman-Duruel ET, Duruel O, Nares S, Stanford C, Tözüm TF (2020). Quantity and Quality of Intraoral Autogenous Block Graft Donor Sites with Cone Beam Computed Tomography. Int J Oral Maxillofac Implants.

[14] Cha HS (2016). Kim JW, Hwang JH, Ahn KM. Frequency of bone graft in implant surgery. Maxillofac Plast Reconstr Surg.

[15] Caracas RE, Manolea HO, Mitrut I, Caraca AM, Salan AI, Draghici MA (2021). Frequency of Bone Augmentation Materials Use in a General Dental Practice. Current Health Sci J.

[16] Ratnayake JTB, Mucalo M, Días GJ (2017). Substituted hydroxyapatites for bone regeneration: A review of current trends: Substituted HA for Bone Regeneration. J Biomed Mater Res B Appl Biomater.

[17] Alswat KA (2017). Gender Disparities in Osteoporosis. Journal of Clinical Medicine Research.

[18] Farr JN, Khosla S (2015). Skeletal changes through the lifespan-From growth to senescence. Nat Rev Endocrinol.

[19] Almeida M, Laurent MR, Dubois V, Claessens F, O'Brien CA, Bouillon R (2017). Estrogens and Androgens in Skeletal Physiology and Pathophysiology. Physiol Rev.

[20] Funato A, Ishikawa T, Kitajima H, Yamada M, Moroi H (2013). A Novel Combined Surgical Approach to Vertical Alveolar Ridge Augmentation with Titanium Mesh, Resorbable Membrane, and rhPDGF-BB: A Retrospective Consecutive Case Series. Int J Periodontics Restorative Dent.

[21] Urban IA, Lozada JL, Jovanovic SA, Nagursky H, Nagy K (2014). Vertical Ridge Augmentation with Titanium-Reinforced, Dense-PTFE Membranes and a Combination of Particulated Autogenous Bone and Anorganic Bovine Bone-Derived Mineral: A Prospective Case Series in 19 Patients. Int J Oral Maxillofac Implants.

[22] Ata-Ali J, Diago JV, Melo M, Bagán L, Soldini MC, Di-Nardo C (2017). What is the frequency of anatomical variations and pathological findings in maxillary sinuses among patients subjected to maxillofacial cone beam computed tomography? A systematic review. Med Oral Patol Oral Cir Bucal.

[23] Mohan N, Wolf J, Dym H (2015). Maxillary sinus augmentation. Dent Clin North Am.

